# Factors Related to Pregnancy and Childbirth and Their Relationship with Exclusive Breastfeeding—A Cross-Sectional Study

**DOI:** 10.3390/nu18030447

**Published:** 2026-01-29

**Authors:** Marcelina Porożyńska, Anna Weronika Szablewska

**Affiliations:** Department of Obstetric and Gynaecological Nursing, Institute of Nursing and Midwifery, Medical University of Gdańsk, Sklodowskiej-Curie 3A, 80-210 Gdansk, Poland

**Keywords:** exclusive breastfeeding, birth satisfaction, lactation, midwifery

## Abstract

**Background/Objectives:** The World Health Organisation (WHO) recommends exclusive breastfeeding for the first six months of life, but exclusive breastfeeding (EBF) rates remain low in many countries, including Poland. Factors related to pregnancy, childbirth and the organisation of care can significantly affect the maintenance of lactation. There is a lack of representative data on these relationships in Poland, which makes it difficult to plan effective support measures. The aim of this study is to analyse the relationship between pregnancy and perinatal factors and exclusive breastfeeding in infants aged 6 to 12 months. **Methods:** This cross-sectional online survey was conducted between April and October 2025. A total of 557 women aged ≥18 years with infants aged 6–12 months participated in the research. Data were collected using a structured questionnaire covering sociodemographic characteristics, the course of pregnancy and childbirth, postpartum complications, early breastfeeding experiences and maternal birth satisfaction assessed using the Polish version of the Birth Satisfaction Scale—Revised (BSS-R). Multivariable logistic regression analysis was used to identify factors associated with exclusive breastfeeding up to six months. **Results:** Exclusive breastfeeding was significantly associated with vaginal delivery, the absence of postpartum complications and a lack of early breastfeeding problems in the first days postpartum, with initial lactation difficulties emerging as its strongest predictor. Most specific pregnancy-related conditions, maternal birth satisfaction and selected recommended hospital practices, including early skin-to-skin contact, were not independently associated with exclusive breastfeeding in the model. **Conclusions:** Exclusive breastfeeding up to six months is primarily determined by factors operating in the immediate perinatal and early postpartum period, particularly postpartum clinical stability and successful early lactation. Targeted support during this critical window may be key to improving exclusive breastfeeding outcomes.

## 1. Introduction

The World Health Organisation (WHO) and other leading health institutions, such as UNICEF and the American Academy of Paediatrics, recommend exclusive breastfeeding for the first six months of a child’s life [1]. Despite clear scientific evidence and recommendations from international organisations, rates of exclusive breastfeeding in many countries, both rich and developing, remain low. This shows a clear discrepancy between recommendations and actual clinical as well as social practice. In Europe, only 25% of women exclusively breastfeed for the first six months of life [2]. The situation is similar in the US—although 81% of women start breastfeeding after giving birth, only 25.5% maintain EBF (Exclusive Breastfeeding) for the first six months of life [3]. In Poland, on the other hand, breastfeeding initiation is high at 97%, but unfortunately, EBF up to six months drops to values between 4 and 6% [4]. Despite the well-known numerous benefits of breastfeeding, exclusive breastfeeding rates are low. Most countries are far from the new EBF target set by the WHO, which states that all countries should achieve at an least 70% EBF prevalence among infants under six months of age by 2030 [5]. Data from studies conducted in Poland between 2014 and 2020 indicate that, despite very high breastfeeding initiation rates (97–99.4%) [6,7,8], the prevalence of exclusive breastfeeding substantially declines over the first months of an infant’s life. Reported rates of exclusive breastfeeding up to six months vary widely, ranging from as low as 4% to 22.4%, depending on the study design, population characteristics and data source [7]. This broad variability underscores the lack of consistent, nationally representative data on exclusive breastfeeding in Poland and highlights the heterogeneity of the existing evidence. Although numerous individual and contextual factors influencing breastfeeding practices have been identified, including demographic, socio-economic, clinical and healthcare-related determinants, the evidence points to a complex interplay of influences rather than single, isolated predictors. In previous studies and systematic reviews, it has been emphasised that biological and healthcare system factors such as mode of delivery, timing of breastfeeding initiation and peripartum care, as well as socio-behavioural factors including maternal education, social support and prenatal care attendance, are associated with exclusive breastfeeding outcomes in diverse settings [9,10]. For example, demographic and clinical variables have been shown to affect exclusive breastfeeding prevalence, while broader determinants, such as breastfeeding knowledge, family and healthcare support and also early breastfeeding practices are recurrently identified as facilitators or barriers to sustained breastfeeding up to six months [11,12].

Factors related to the course of pregnancy and childbirth play an important role in the success of breastfeeding. Both medical aspects (method of delivery and perinatal complications) and organisational or psychosocial factors (skin-to-skin contact, rooming-in, access to a lactation consultant, social support and the mother’s intentions) are highlighted in the literature on the subject [13,14,15,16,17].

Despite extensive research on factors supporting breastfeeding, evidence regarding the determinants of exclusive breastfeeding among Polish women remains fragmented and inconsistent. The increasing medicalisation of pregnancy and childbirth, changing social demands and the growing presence of social media may also influence women’s feeding choices, but these relationships remain unclear. For this reason, it is important to re-examine the factors related to pregnancy and the perinatal period that are associated with exclusive breastfeeding in order to gain a more accurate understanding of the current situation and develop more effective methods of supporting breastfeeding. Additionally, in the majority of available studies, selected determinants have been examined in isolation, whereas fewer analyses have been focused on the assessment regarding the relative contribution of medical, perinatal and early lactation-related factors within a single multivariable framework.

The aim of this study is to analyse the relationship between pregnancy and perinatal factors and exclusive breastfeeding in infants aged six to 12 months. The study is focused on assessing how the course of pregnancy, mode of delivery, perinatal complications and maternal birth experience may influence a mother’s decision to initiate and maintain exclusive breastfeeding, in accordance with WHO recommendations.

The results obtained may form the basis for developing more effective strategies to support and promote breastfeeding, including educational programmes for parents, specialist training for medical staff and organisational solutions conducive to creating a breastfeeding-friendly environment.

## 2. Materials and Methods

### 2.1. Study Design

This trial was a quantitative, cross-sectional observational study conducted among 557 mothers of infants aged six to 12 months. In Poland, ethnic minorities constitute a relatively small part of the population, therefore no distinction was made between ethnic groups. The study design was planned and described in accordance with the STROBE guidelines for cross-sectional studies [18]. All procedures were conducted in accordance with the principles of the 1964 Declaration of Helsinki of the World Medical Association on research involving human subjects and were approved by the Bioethics Committee of the Medical University of Gdańsk (Decision No. KB/19/2025).

### 2.2. Study Setting

The study was conducted using an online diagnostic survey, which participants completed independently. The data collection and participant recruitment period lasted from April to October 2025. The research was carried out using the Computer-Assisted Web Interview method, which made it possible to reach a wide and diverse group of respondents. A link to the anonymous online survey was shared via parenting websites and services, thematic discussion forums, support groups for mothers of infants and social media platforms. Thanks to this approach, women from different regions of Poland were able to participate in the study, which allowed for the collection of highly diverse and representative data. A total of 1121 individuals opened the online survey link. Of these, 557 respondents completed the questionnaire to an extent sufficient for inclusion in the analyses, while 564 respondents did not proceed to subsequent questions or submitted incomplete questionnaires, corresponding to a non-completion rate of approximately 50.3%. Only questionnaires with complete data for variables included in the regression model were retained for statistical analysis; cases with missing values were excluded listwise.

### 2.3. Participants

Before completing the questionnaire, participants were given detailed information about the study and provide their informed consent to participate in the study via electronic form. Open online recruitment was used. A total of 557 women took part in the analysis. The characteristics of the study population are presented in Table 1.

### 2.4. Inclusion and Exclusion Criteria

The inclusion criteria were women aged 18 years or older who had infants aged 6–12 months, were exclusively breastfeeding, and gave their informed consent for participation, were included in the study. The research was carried out in electronic form and consisted of ticking a box to agree to participate in the study and reading the information, which opened the form. Exclusion criteria included Mothers under the age of 18, and of infants younger than 6 months or older than 12 months, as well as participants who did not speak Polish to a degree sufficient to complete the questionnaire independently, were excluded from the study. Women reporting cognitive difficulties that prevented them from completing the questionnaires were also omitted from participation. Fathers and other legal guardians of infants were not invited to participate in the study because the aim of the project was to analyse attitudes and psychosocial conditions specific to the experiences of mothers during the postpartum and breastfeeding periods. The above exclusion criteria were presented in the study information, along with detailed instructions, definitions and information allowing respondents to be provided with comprehensive information. An additional safeguard was provided by the survey questions, which allowed for the exclusion of participants (e.g., when reporting medical conditions or gender).

In this study, the independent variables were:

- pregnancy-related medical factors: gestational diabetes, hypothyroidism, hypertension and/or preeclampsia, threatened preterm labour and other pregnancy complications;
- birth-related factors: mode of birth (vaginal birth, planned caesarean section, unplanned caesarean section), postpartum complications (postpartum haemorrhage, poor neonatal condition, use and type of labour analgesia);
- early postnatal practices: lack of skin-to-skin contact after birth, delayed initiation of breastfeeding (>2 h after birth), early lactation difficulties during the first days postpartum.
- birth experience, measured using the Birth Satisfaction Scale-Revised (BSS-R), allowing for the assessment of a woman’s satisfaction with the course of labour.

The dependent variable was the women’s responses regarding breastfeeding practices, in particular:

- maintaining exclusive breastfeeding until the child was six months old;
- total duration of breastfeeding until the child was 12 months old.

### 2.5. Data Collection Tools

Data was collected from the respondents using a questionnaire consisting of two parts:

#### 2.5.1. Original Questionnaire

The first part of the questionnaire contained closed and open questions concerning basic socio-demographic data (including age, place of residence, education, professional and economic status), obstetric history (number of pregnancies, births, miscarriages), information related to breastfeeding and the mother’s health.

Questions referring to medical conditions during pregnancy, childbirth and the early postpartum period were accompanied by brief explanatory notes and examples to ensure a consistent understanding of the terms used. In particular, “other pregnancy complications” referred to less common or non-specific medical conditions occurring during pregnancy (e.g., infections, bleeding, placental abnormalities), while postpartum complications included conditions occurring during the puerperium, such as postpartum haemorrhage, retained placental tissue requiring uterine curettage, problems with the wound healing process and maternal infections.

Early breastfeeding problems were defined as difficulties occurring in the first days postpartum and included issues such as problems with infant latch, nipple pain, perceived insufficient milk supply and delayed onset of lactogenesis II.

#### 2.5.2. Birth Satisfaction Scale-Revised (BSS-R)

The second part of the questionnaire was a Polish adaptation of the Birth Satisfaction Scale-Revised (BSS-R) [19]. This scale was developed by Hollins Martin and Fleming to assess women’s satisfaction with the course of childbirth. The BSS-R consists of 10 items rated on a five-point Likert scale (from ‘strongly disagree’ to ‘strongly agree’), which form three subscales:

- Quality of care (e.g., support from medical staff);
- Stress and anxiety related to childbirth (e.g., feeling loss of control);
- Psychological factors (e.g., satisfaction with one’s own coping skills).

In this study, only the overall result—the predictor—into account, without distinguishing between subscales.

Higher scores indicate greater satisfaction with the birth experience. The tool has good psychometric properties, and its Polish adaptation has been found to be highly reliable, as assessed by Cronbach’s alpha (α = 0.79–0.87 depending on the subscale).

### 2.6. Sample Size

A total of 557 observations with complete data were included in the logistic regression analysis, while cases with missing values for any of the variables included in the model were excluded listwise. The sample size was determined based on the available population reached through online recruitment during the study period. A post hoc power analysis indicated that the final sample provided sufficient power (>80%) to detect small-to-moderate effect sizes in logistic regression analyses with multiple predictors. Given the number of observations and approximately 17 predictors in the model, the results of the logistic regression can be considered reliable. According to the commonly cited rule of at least 10 events per predictor, the sample size ensured stable estimation of regression coefficients and maintained the validity of the model.

### 2.7. Bias

Due to the online, self-reported nature of the study, the potential for selection bias cannot be excluded, as participation may have been more likely among women with higher health awareness or interest in breastfeeding. Recall bias is also possible, particularly regarding early postpartum experiences. In addition, some perinatal and medical variables may be subject to misclassification due to maternal interpretation of clinical terms. To mitigate this, the study included only mothers of infants aged 6–12 months, reducing long-term recall error. Additionally, standardised and validated instruments (BSS-R) were used where applicable.

### 2.8. Statistical Analysis

Multivariable logistic regression analysis was conducted to examine factors associated with exclusive breastfeeding up to six months of age. Exclusive breastfeeding was coded as ‘1’, whereas partial breastfeeding or formula feeding was coded as ‘0’. All clinically and theoretically relevant variables, including pregnancy-related medical conditions, delivery and postpartum factors, early breastfeeding practices, birth satisfaction (BSS-R score) and selected socio-demographic characteristics, were entered simultaneously into the model to assess their independent effects. The adequacy of the model fit was evaluated using the Hosmer–Lemeshow goodness-of-fit test. Odds ratios (Exp(B)) with 95% confidence intervals were calculated to quantify the strength and direction of associations. Statistical significance was set at *p* < 0.05. All analyses were performed using IBM SPSS Statistics, version 29.

## 3. Results

The overall regression model was statistically significant (χ^2^ = 92.029, df = 17, *p* < 0.001), indicating that the included predictors significantly differentiated between women who maintained exclusive breastfeeding and those who did not. The model allowed to demonstrate moderate explanatory power, with Cox and Snell’s R^2^ of 0.153 and Nagelkerke’s R^2^ of 0.231. The Hosmer-Lemeshow goodness-of-fit test was not statistically significant (χ^2^ = 2.254, df = 8, *p* = 0.972), suggesting good agreement between observed and predicted outcomes.

The detailed results of the multivariable logistic regression analysis are presented in Table 2.

Results from the logistic regression analysis can be found in Table 2.

Most health issues linked to pregnancy, such as gestational diabetes, high blood pressure due to pregnancy, thyroid problems, preeclampsia and risks of early labour, did not show a significant link with exclusive breastfeeding for six months. However, having other unspecified pregnancy issues was connected with a lower chance of practicing exclusive breastfeeding (Table 2).

Factors related to delivery showed various significant correlations. A vaginal delivery and no complications after giving birth were positively related to exclusive breastfeeding, while receiving anaesthesia during childbirth did not have such an association (Table 2).

Initial breastfeeding actions, such as skin-to-skin contact during the first two hours after birth and the timing of the first breastfeeding session, did not significantly relate to exclusive breastfeeding. On the other hand, early experiences with lactation were identified as the strongest predictor in the analysis. Mothers who did not encounter any breastfeeding or lactation difficulties in the early postpartum days were significantly more likely to continue exclusive breastfeeding (Table 2).

The overall score on the Birth Satisfaction Scale-Revised (BSS-R) did not show a meaningful association with outcomes of exclusive breastfeeding. Likewise, none of the socio-demographic factors considered for control—such as maternal age, educational level, self-assessed financial situation and number of previous births—indicated statistically significant connections with exclusive breastfeeding for up to six months (Table 2).

## 4. Discussion

In the present study, evidence is provided that allows to note exclusive breastfeeding up to six months of age is primarily associated with factors operating at the proximal, physiological and early postnatal levels of a multilevel determinants framework. Within this perspective, the associations were observed for early lactation success, absence of postpartum complications and vaginal delivery, whereas birth satisfaction and selected recommended hospital practices did not show independent associations in the model. These findings suggest that, in the context of medically problematic pregnancies and births, direct biological and functional determinants may outweigh more distal psychosocial or organisational factors when predicting longer-term exclusive breastfeeding.

The findings of the present study could be interpreted within a multilevel ecological model of breastfeeding determinants, informed by Bronfenbrenner’s ecological systems theory [20] and conceptual frameworks proposed by the World Health Organization [21]. These approaches conceptualise exclusive breastfeeding as an outcome shaped by interacting influences operating at multiple levels, ranging from proximal biological and behavioural factors to institutional, psychosocial and broader contextual conditions. Within this framework, our results indicate that factors located at the micro-level of the system, particularly early lactation functioning and immediate postpartum health, play a dominant role in sustaining exclusive breastfeeding up to six months of age. In contrast, more distal determinants, such as birth satisfaction or isolated hospital practices, did not demonstrate independent associations when considered alongside biomedical and early postnatal factors. This pattern suggests that, in populations characterised by a high degree of medicalisation of pregnancy and childbirth, the effectiveness of psychosocial and institutional influences may be contingent upon the successful establishment of early physiological processes underlying lactation.

In the present study, specific pregnancy-related conditions, including gestational diabetes mellitus and hypertensive disorders of pregnancy, were not independently associated with exclusive breastfeeding up to six months. In contrast, the presence of other, non-specific pregnancy complications significantly reduced the likelihood of maintaining exclusive breastfeeding. This finding indicates that the overall clinical burden of pregnancy, rather than individual diagnostic categories, may be more relevant for long-term breastfeeding outcomes. In previous studies, it has been demonstrated that gestational diabetes mellitus and hypertensive disorders of pregnancy are associated with early breastfeeding difficulties, such as delayed onset of lactogenesis, perceived insufficient milk supply and an increased risk of early breastfeeding discontinuation [22,23,24].

The lack of independent associations for these specific diagnoses in the adjusted model of the present study does not contradict this evidence, but rather indicates that their influence on exclusive breastfeeding may be heterogeneous and context-dependent, and potentially overshadowed by the cumulative impact of multiple or less well-defined pregnancy complications captured in the category of other pregnancy-related conditions.

The association between mode of delivery and exclusive breastfeeding observed in this study is consistent with a substantial body of previous research. Vaginal birth has repeatedly been linked to earlier initiation of breastfeeding and higher rates of exclusive breastfeeding, while caesarean section is associated with delayed lactogenesis, difficulties in early mother–infant interaction and increased supplementation. In prospective cohort studies, it is indicated that for women who deliver by caesarean section, it is less probable that they will initiate breastfeeding within the first hour of life and are more likely to discontinue exclusive breastfeeding during the first months postpartum [25,26].

However, birth satisfaction was not independently associated with exclusive breastfeeding in the analysis. This result may reflect mediation pathways, whereby the effect of childbirth experience on breastfeeding operates indirectly through early lactation success or postpartum recovery. It is also possible that birth satisfaction is more strongly related to breastfeeding initiation or short-term persistence than to exclusive breastfeeding at six months when biomedical and early functional factors are taken into account. Importantly, evidence from intervention studies demonstrates that structured breastfeeding support provided by healthcare professionals improves exclusive breastfeeding rates, underscoring that psychosocial and care-related factors remain relevant at the system level, even if a single satisfaction measure does not predict long-term outcomes independently [13,15,27].

One institutional practice shown to partially mitigate the adverse breastfeeding consequences of caesarean delivery is early skin-to-skin contact. In a recent meta-analysis, it was demonstrated that early skin-to-skin contact after caesarean section shortens the time to first breastfeeding by approximately 52 min, increases the likelihood of breastfeeding within the first two hours by 95%, and improves exclusive breastfeeding rates at hospital discharge; however, it did not significantly extend exclusive breastfeeding duration at one month or later [28]. Nevertheless, implementation remains limited: data from maternity hospitals in central Poland show that immediate skin-to-skin contact after caesarean section occurs in only 11.73% of cases and is often brief, lasting only a few minutes [29]. These findings are particularly relevant in the Polish context, where caesarean section rates are among the highest in Europe. National Health Fund (NFZ) reporting based on routine healthcare data indicates that caesarean sections account for approximately 47–48% of births in Poland, far exceeding international recommendations that suggest an optimal national caesarean rate of 15–20% [30]. Within an ecological framework, the high prevalence of operative birth represents a structural, system-level exposure that may shift breastfeeding outcomes at the population level, potentially contributing to persistently low exclusive breastfeeding rates despite high.

An important finding of this study is the strong association between the absence of postpartum complications and exclusive breastfeeding. In the present analysis, postpartum complications referred to conditions occurring during the puerperium, including postpartum haemorrhage, uterine curettage related to retained placental tissue, perineal infections and maternal infectious complications. Such conditions may directly disrupt the establishment of breastfeeding through delayed maternal recovery, increased pain, limited mobility, mother–infant separation and—importantly—through biological interference with lactogenesis II, for example, due to retained placental fragments, inflammation or significant blood loss [31]. This finding supports the interpretation that the cumulative physiological burden of the postpartum period, rather than isolated prenatal diagnoses, may play a role in sustaining exclusive breastfeeding. From a physiological perspective, postpartum morbidity can impair the normal endocrine cascade required for the timely onset of copious milk secretion, thereby increasing the risk of early supplementation and subsequent discontinuation of exclusive breastfeeding initiation prevalence.

The most pronounced association observed in the present study concerned early lactation problems during the first days postpartum. Women who did not report early breastfeeding difficulties had markedly higher odds of maintaining exclusive breastfeeding up to six months. This finding is in line with qualitative and mixed-methods evidence identifying early pain, latch difficulties, perceived insufficient milk supply and delayed onset of lactogenesis as central reasons for supplementation and early cessation of exclusive breastfeeding [22].

These findings highlight the importance of prioritising interventions that minimise perinatal and postpartum complications and ensure immediate, skilled support for early lactation, particularly in settings with high rates of medicalised birth. Rather than focusing solely on isolated practices, comprehensive care models that address cumulative clinical burden and protect early lactation functioning may offer greater potential to improve exclusive breastfeeding rates.

Limiting the research group to infants between six and 12 months of life probably minimised long-term memory mistakes regarding the main outcome measure (exclusive breastfeeding), but it might have affected the precision of reporting on less obvious perinatal occurrences. Additionally, mothers’ reflections on the reasons behind their experiences could have been swayed by whether they successfully continued breastfeeding, which might introduce personal bias. These constraints are typical of retrospective observational studies and must be considered when analysing our findings.

### Strengths and Limitations

Several strengths and limitations of this study should be considered. The strengths include the relatively large sample size, the inclusion of a broad range of biomedical, perinatal and psychosocial variables within a single analytical model, and the use of a validated instrument to assess birth satisfaction. The limitations include the cross-sectional design, which precludes causal inference, reliance on self-reported data and potential selection bias related to online recruitment. Recall bias cannot be excluded, although restricting the sample to mothers of infants aged 6–12 months likely reduced long-term recall error. The use of an online, self-administered questionnaire limited opportunities to clarify ambiguous responses; nevertheless, previous research suggests that data collected via standardised online surveys can be comparable in reliability to those obtained through face-to-face methods, particularly when validated instruments are used.

An additional limitation concerns the retrospective self-reporting of selected perinatal and medical variables, such as neonatal condition, type of labour analgesia and timing of breastfeeding initiation. These variables may be affected by maternal misperception or imperfect recall, and the study design did not allow for cross-validation against medical records. Consequently, exposure misclassification cannot be excluded, which—if largely non-differential—may have attenuated true associations.

An additional limitation concerns the sampling strategy and sample composition. Open online recruitment is known to favour participation of women with higher educational attainment, greater digital literacy and higher health awareness. Accordingly, women with higher education were overrepresented in the present sample, while mothers with lower educational levels, limited internet access or lower health literacy, may be underrepresented. This pattern is common in online studies conducted in perinatal populations and may limit the generalisability of the findings to socioeconomically more disadvantaged groups. Moreover, women who are active in online parenting communities and motivated to engage in health-related research may differ systematically from those who do not participate in such platforms. Despite these limitations, the study provides valuable insights into the relative importance of multilevel determinants of exclusive breastfeeding in a national context where such integrated analyses remain scarce.

Future research should employ longitudinal designs to explore causal pathways and mediation effects, particularly the role of early lactation problems as a potential mediator between medical factors and long-term breastfeeding outcomes. More detailed measurements of early breastfeeding practices and cumulative clinical burden may further refine the understanding of mechanisms identified in this study.

## 5. Conclusions

In this study, it is demonstrated that exclusive breastfeeding up to six months is primarily determined by factors operating in the immediate perinatal and early postpartum period, rather than by isolated pregnancy-related diagnoses or individual recommended hospital practices. In particular, the absence of postpartum complications and early lactation difficulties emerged as the strongest predictors of sustained exclusive breastfeeding, while most specific pregnancy-related conditions and maternal birth satisfaction did not independently predict long-term breastfeeding outcomes when analysed within a comprehensive model.

These findings underscore the importance of viewing breastfeeding as a dynamic, multilevel process, in which cumulative clinical burden and early physiological stability play a decisive role. The results suggest that interventions aiming to improve exclusive breastfeeding rates should prioritise prevention and effective management of postpartum complications and ensure intensive, skilled support during the critical early days of lactation, especially for women experiencing medically complex pregnancies or births.

By integrating biomedical, perinatal and psychosocial factors within a single analytical framework, this study contributes to a more nuanced understanding of breastfeeding determinants in the Polish context and highlights the need for future longitudinal research to clarify causal pathways linking pregnancy, childbirth, early lactation and long-term breastfeeding outcomes.

## Figures and Tables

**Table 1:** Characteristics of the study group.

Variable	Category	N/M	%/SD
Age		32.33	5.03
Education	Secondary	84	15.1
	Higher	466	83.7
	Vocational	7	1.3
Place of residence	City ≤ 100,000 inhabitants	146	26.2
	City > 100,000 inhabitants	262	47.0
	Rural	149	26.8
Self-assessed material status	Very good	142	25.5
	Good	332	59.6
	Moderate	81	14.5
	Poor	1	0.2
	Very poor	1	0.2
Self-assessed housing conditions	Very good	261	46.9
	Good	239	42.9
	Moderate	54	9.7
	Poor	3	0.5
Pregnancy complications or pre-existing conditions	No	364	65.4
	Yes	193	34.6
Postpartum complications	No	110	19.7
	Yes	447	80.3
Parity	No	340	61.0
	Yes	217	39.0
Mode of birth	Vaginal delivery	346	62.1
	Caesarean section	211	37.9
BSS-R		22.95	3.30
Was continuous 2 h skin-to-skin contact maintained immediately after birth?	No, it was interrupted without a medical reason	73	13.1
	No, due to the mother’s medical condition	52	9.3
	No, due to the infant’s medical condition	64	11.5
	Yes	368	66.1
Timing of first breastfeeding initiation	More than 2 h after birth	118	21.2
	Breastfeeding was not initiated	6	1.1
	Within 2 h after birth	433	77.7

**Table 2:** Multivariable logistic regression analysis of factors associated with exclusive breastfeeding up to six months of age.

Variable	B	SE	Wald	*p*	OR	95% CI OR
BSS-R (total score)	−0.022	0.034	0.428	0.513	0.978	0.916–1.045
Gestational diabetes	−0.372	0.293	1.613	0.204	0.689	0.388–1.224
Pregnancy-induced hypertension (PIH)	−0.462	0.381	1.473	0.225	0.630	0.299–1.329
Hypothyroidism	0.234	0.250	0.872	0.350	1.263	0.773–2.063
Preeclampsia	−0.124	0.715	0.030	0.863	0.884	0.218–3.588
Threatened preterm labour	0.273	0.430	0.404	0.525	1.314	0.566–3.049
Other pregnancy complications	−0.741	0.293	6.403	**0.011**	0.476	0.268–0.846
Mode of birth (vaginal delivery vs. caesarean section) ^A^	0.608	0.272	4.998	**0.025**	1.837	1.078–3.130
Use of anaesthesia	−0.081	0.282	0.081	0.775	0.923	0.531–1.604
No early lactation problems	2.755	0.610	20.400	**<0.001**	15.720	4.756–51.959
No postpartum complications	0.777	0.269	8.368	**0.004**	2.174	1.285–3.681
Skin-to-skin contact within 2 h	0.100	0.260	0.147	0.701	1.105	0.664–1.840
First breastfeeding ≤ 2 h ^B^	−0.039	0.288	0.018	0.892	0.962	0.547–1.690
Maternal age (control)	−0.024	0.025	0.918	0.338	0.976	0.930–1.025
Education level (control) ^C^	0.307	0.299	1.055	0.304	1.359	0.757–2.440
Financial status (control) ^D^	0.332	0.288	1.330	0.249	1.393	0.793–2.448
Parity (control) ^E^	0.275	0.254	1.172	0.279	1.317	0.800–2.168

Note: Categories were coded as follows: A = vaginal delivery (1) and caesarean section (0); B = within two hours (1) and more than two hours (0); C = higher education (1) and other than higher education (0); D = very good/good financial situation (1) and moderate/poor financial situation (0); E = primiparous (1) and multiparous (0). Abbreviations: OR—odds ratio; CI—confidence interval; BSS-R—Birth Satisfaction Scale-Revised. Statistical significance: *p* < 0.05 (bolded).

## Data Availability

The original contributions presented in the study are included in the article; further inquiries can be directed to the corresponding author.

## Abbreviations

The following abbreviations are used in this manuscript:

WHO	World Health Organization
EBF	Exclusive breastfeeding
BSS-R	Birth Satisfaction Scale—Revised
UNICEF	United Nations International Children’s Emergency Fund
WMA	World Medical Association
CAWI	Computer-assisted web interview
STROBE	Strengthening the Reporting of Observational Studies in Epidemiology
NFZ	National Health Fund
OR	Odds ratio
